# Biochemical osteomalacia during pregnancy or lactation: an observational study in Sweden

**DOI:** 10.1186/s12884-026-08848-1

**Published:** 2026-02-24

**Authors:** Thomas Torstensson, Per Kristiansson, Paul Kalliokoski

**Affiliations:** Department of Public Health and Caring Sciences, Family Medicine and Preventive Medicine Section, Uppsala University, Uppsala, Box 564, SE-751 22 Sweden

**Keywords:** Women’s Health Issues, Pregnancy, Antenatal Care, Osteomalacia, Vitamin D

## Abstract

**Background:**

Studies of osteomalacia during pregnancy and lactation are scarce. This is likely due to the hazards of performing X-ray in pregnancy, the inconvenience of the gold standard diagnostic procedure of bone marrow biopsy with histomorphometry and the lack of these resources in maternity health care. Another important reason is the lack of international consensus on how to diagnose osteomalacia clinically. Osteomalacia needs attention due to the limited number of modern studies on its prevalence and globalization, especially in some populations where migration to high latitudes with poor light conditions, as in Scandinavia, can heighten its prevalence. The aim of this study was to determine the prevalence of biochemical osteomalacia during pregnancy or lactation among Somali and Swedish women living in Sweden.

**Methods:**

This was an observational cohort study of 71 Swedish and 52 Somali pregnant or lactating women. Blood samples, self-report questionnaires and physical examination data were collected in late spring. The diagnostic criteria for biochemical osteomalacia were serum levels of a 25-hydroxy vitamin D_3_ (25(OH)D) concentration < 30 nmol/L, a parathyroid hormone (PTH) concentration > 6.9 pmol/L and an alkaline phosphatase (ALP) concentration > 1.8 ukat/L. The presence of clinical symptoms (grip weakness, leg weakness, a positive Trendelenburg test, fatigue, and pain) was used to reaffirm the diagnosis.

**Results:**

The diagnostic criteria for biochemical osteomalacia were met by one Swedish woman 1/71 (1.4%) and 18/52 (34.6%) of all Somali women, of whom 1/71 (1.4%) and 16/52 (30.8%), respectively, had signs or symptoms reaffirming the diagnosis.

**Conclusions:**

Women of Somali origin living at high altitudes are at high risk for vitamin D deficiency osteomalacia, but Swedish women may also suffer from this disease. These findings call for further studies on the prevalence of vitamin D deficiency, especially among groups at risk of vitamin D deficiency, because of the hidden disease burden. Establishing internationally accepted criteria for a noninvasive clinical diagnostic procedure for osteomalacia is imperative.

**Trial registration:**

ClinicalTrials.gov Identifier: NCT02922803. Date of registration: September 28, 2016.

**Supplementary Information:**

The online version contains supplementary material available at 10.1186/s12884-026-08848-1.

## Background

Signs of osteomalacia (“bone softening”) have been observed and described for thousands of years, for example, by the Greek obstetrician Soranus of Ephesos, who was active in Rome around the year 150 A.D. The disease has been systematically described since the time of industrialization [1]. The prevalence of osteomalacia is difficult to define since the condition is often asymptomatic. In bone biopsy studies of femoral fracture patients, the prevalence of osteomalacia increases with age, and osteomalacia occurs in approximately 30% of the patients [2, 3].

There are four pathogenic mechanisms involved in the development of osteomalacia, among which vitamin D deficiency is the most common. The clinical manifestations of vitamin D deficiency osteomalacia include diffuse bone pain and tenderness, muscle weakness, fatigue and fragility fractures [4]. Vitamin D is a circulating hormone that regulates mineral and skeletal homeostasis, including calcium metabolism, with particular challenges during pregnancy and lactation. The concentration of 25-hydroxy vitamin D_3_ (25(OH)D) in serum is an indicator of vitamin D status [5]. Low levels of 25(OH)D and low calcium lead to osteomalacia [6, 7]. Extrinsic causes of vitamin D deficiency are decreased sunlight exposure, including covering clothing and dark skin pigmentation, which make immigrants from lower to higher northern latitudes particularly vulnerable [8, 9]. In addition, the prevalence of indoor lifestyles has increased among people in Sweden as well as in many other Western countries. Recent directions toward computer and screen use, both for work and leisure, may have contributed to lower sun exposure and vitamin D levels.

The most accurate method for diagnosing vitamin D deficiency osteomalacia in patients is iliac crest biopsy, while distinctive noninvasive testing for identifying osteomalacia is lacking. However, biochemical osteomalacia criteria are proposed according to serum levels of 25(OH)D, alkaline phosphatase (ALP), and parathyroid hormone (PTH) [10]. To reaffirm the biochemical diagnosis, the presence of clinical symptoms should be considered. Spontaneous fractures, hypocalcemia and seizures are signs of more advanced and severe disease [11].

During pregnancy and lactation, female physiology adapts to meet the added nutritional demands of fetuses and neonates. During pregnancy, the efficiency of intestinal calcium absorption doubles, whereas during lactation, the maternal skeleton is resorbed to provide calcium for milk [12]. These mechanisms include increased calcitriol (1,25(OH)_2_D) production during pregnancy and increased parathyroid hormone-related protein (PTHrP) production during pregnancy and lactation. This normally entails low levels of PTH production throughout both pregnancy and lactation [13–17]. Thus, PTH levels are comparable in pregnancy and lactation [12].

Studies of osteomalacia during pregnancy and lactation are scarce when searching databases such as PubMed; therefore, we cannot present many modern references in the literature. There may be few studies due to the hazards of X-ray examinations during pregnancy, the invasiveness and limited feasibility of the gold standard diagnostic procedure, bone biopsy with tetracycline labeling and histomorphometry, given the contraindication of tetracyclines in pregnancy due to fetal skeletal and dental deposition, and the lack of access to these resources within maternity and primary health care, particularly in low-resource settings. Another important reason is likely the absence of an international consensus on how to diagnose osteomalacia clinically using non-invasive and non-radiation-based methods. This study is a response to the call for further studies to explore the prevalence of solar and nutritional osteomalacia in various populations to determine whether there is a hidden disease burden [10].

## Methods

The aim of this study was to determine the prevalence of osteomalacia during pregnancy and lactation among Somali and Swedish women living in Sweden.

### Study design

This was an observational cohort study of pregnant and lactating Somali and Swedish women. Blood samples, self-reported questionnaire data, and physical examination findings were collected within a few weeks in late May and early June.

### Setting

A primary healthcare clinic in Borlänge, a town in mid-Sweden at latitude 60°N. At that time, Borlänge had approximately 50 000 inhabitants, which were mostly populated by people of Swedish origin. However, since the beginning of the year 2000, there has been a successive migration to Borlänge of Somali women, who, at that time, amounted to approximately 2000 women of fertile age.

### Participants

**Fig. 1:**
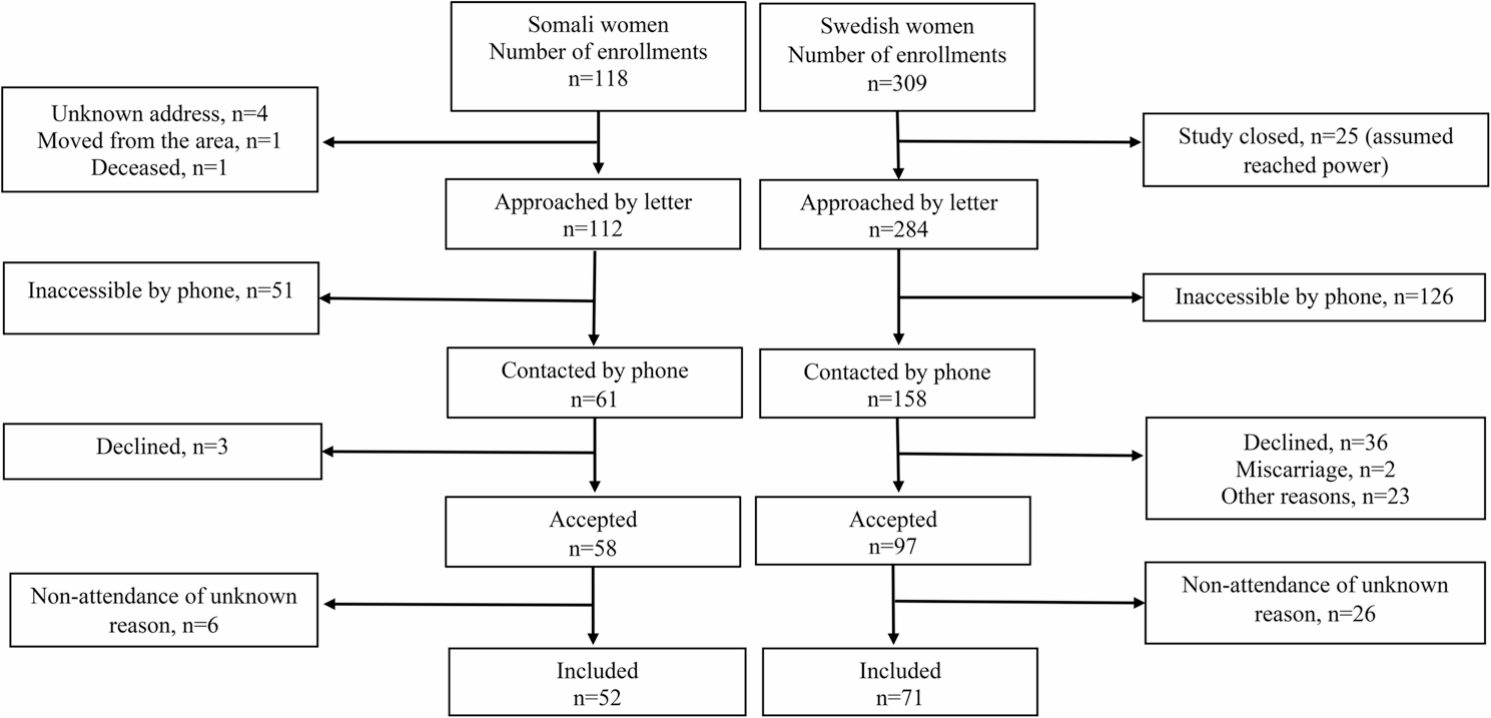
Flowchart of participating women born in Somalia and Sweden. The participants were recruited from September 2008 to June 2010

Women registered at the mother health care clinic in Borlänge from September 2008 to June 2010 (21 months) were eligible for the study. All women retrospectively included in the study were either pregnant or tentatively lactating up to one year after delivery. One hundred eighteen Somali women were eligible during the period of enrollment, and 112 were approached (Fig. 1). Somali women living in Borlänge in general wear veiling clothing apart from having strong skin pigmentation, making them prone to reduced sun exposure and vitamin D deficiency. Three hundred and nine Swedish women were randomly selected from a retrograde ordered sample list.

All participants were enrolled in standard Swedish antenatal care, which includes routine screening for major conditions, i.e. hemoglobin, blood pressure, urine dipstick, plasma glucose, and TSH, with referral to specialist care if abnormalities were detected. 

### Recruitment procedure

An invitation letter with study information was sent to the women. One week after the letter was sent, an assistant Somali nurse or one research medical doctor (PKa) attempted to reach the woman by telephone. The telephone numbers were searched from public databases on the Internet (Eniro^®^). Approximately half of the women approached with a letter were inaccessible by phone. After providing oral information about the study, 58 Somali women and 97 Swedish women agreed to participate. Women who agreed to participate were scheduled for written consent, blood sampling (fasting) and appointment for a study visit. Blood samples were centrifuged, kept cool on ice and secured from ultraviolet light, transported to the laboratories and examined within a few days. The biochemical panel for osteomalacia assessment included 25(OH) D, PTH, ALP, and phosphate, alongside routine antenatal measures; these were analysed as part of our non-invasive diagnostic protocol.

Fifty-two Somali and 71 Swedish women were included. The inclusion criteria were being ≥ 18 years of age, being registered at the mother health care clinic and being a resident of Region Dalarna. The exclusion criteria were birthplace other than Sweden or Somalia and severe mental or somatic disorders.

At the study visit, the women completed a questionnaire, and a physician performed a physical examination. The questionnaire included items on sociodemographic variables, sun exposure, physical activity, intake of milk, fat fish, cheese, and mineral/vitamin supplements, current pain and fatigue. It also covered medical history including endocrine and neurological disorders and ongoing medication including corticosteroids. The physical examination included measurements of muscle strength and was performed by either of two researchers. Height and weight were measured by assistant nurses. Delivery records were reviewed to verify absence of severe untreated endocrine disease.

The questionnaire was written in Swedish and is described in an earlier study published in BMC Pregnancy and Childbirth 2013 [18]. The Swedish women completed the questionnaire in private. The Somali women completed the questionnaire with help from professional interpreters. Following completion, the researchers checked whether all questions had been answered completely, and the women had the opportunity to ask about the meaning of the questions, if necessary.

### Outcome

Diagnosis of biochemical osteomalacia reaffirmed by signs and symptoms (no/yes) among women of Somali and Swedish origin living in Sweden.

### Variables of interest

The included variables, their definitions and scaling were as follows:

*Veiled clothing*, at least long-sleeved arms and legs and covered hair and neck was used during the summer (no/yes).

*Outdoors*, ≥ 30 min every day during the summer (no/yes).

*Sunbathing* face, neck and forearms during summer in Sweden (no/yes).

*Sun holiday* in winter, sunbathing at lower latitudes (no/yes).

*Time in Sweden* (years).

*Time to inclusion*, weeks of gestation or weeks from delivery to the date of study inclusion.

*Pregnant* (no/yes), self-reported.

*Weeks of gestation*, self-reported completed weeks since the last menstrual period or by ultrasound assessment.

*The time after delivery* was defined as the number of weeks from the self-reported date of delivery until the study visit.

*In lactation* (no/yes) lasted until one year after childbirth.

*Parity* was defined as the self-reported number of previous births.

*Age* (years) from the date of birth to the date of the study visit.

*Education* (number of years), self-reported.

*Weight* before pregnancy, self-reported (kg).

*Height* (cm) was measured by a wall-mounted tape measure without shoes to the nearest centimeter.

*Physical activity*, ”Do you exercise (walking at least 30 min or home exercises)? 0–2 times/w (= 0), > 2 times/w [1]”.

*Milk consumption*, “Approximately how many decilitres or glasses of milk, sour milk or yoghurt do you drink/eat every day? Answers in decilitres, or glasses”.

*Fat fish consumption*,* “*How many times per week do you eat salmon or other fatty fish? Never (= 0), 1–2 times (= 1), 3 times or more (= 3)”.

*Cheese consumption*, “How often do you eat cheese? Answers were ticked in boxes: never (= 0), 1–2 times/w (= 2), 3–5 times/w (= 4) and 6–7 times/w (= 6)”.

*Grip strength* and peak grip strength, defined as the highest value of three trials in each hand, were measured in Newtons using a hand dynamometer (Grippit^®^) [19, 20].

*Grip weakness* was defined as less than − 2 standard deviations [19] of peak grip strength measured with a hand dynamometer.

*Squat*, squat and rise once. A minimum 90° angle between the thigh and lower leg was used and scaled as 0 = without difficulty, 1 = with difficulty (e.g., support by hands on knees), 2 = with great difficulty (e.g., support by hands on furniture) or unable to arise from the squatted position. *Leg weakness* was defined as the inability to perform one squat without difficulty (scores of 1 and 2), corresponding to the 75th percentile.

*Trendelenburg’s test* involved standing on one leg, with one hand high up on a wall, and lifting the opposite leg for 30 s [21], scaled as follows: 0 = maintained pelvis horizontal to the floor, 1 = unable to maintain pelvis horizontally to the floor, 2 = unable to stand on one leg because of muscle weakness.

A positive Trendelenburg test (a sign of weakness of the hip abductor) was given a score of 1 or 2, corresponding to the 90th percentile.

*Fatigue*, experienced tiredness during the past month marked on a 100 mm long visual analog scale with not tired at all = 0 mm and unbearably tired = 100 mm.

*Pain intensity*, experiencing pain intensity now, regardless of location, marked on a 100 mm long visual analog scale with no pain at all = 0 mm and unbearable pain = 100 mm.

*S-25(OH)D* was measured using a Liaison 25 OH vitamin D total assay (DiaSorin, Stillwater, MN, USA) in a certified clinical chemistry laboratory at University Hospital, Uppsala, Sweden. The lowest detectable level was < 10 nmol/L, which was replaced with 9 nmol/L in the calculations. The method detects both 25(OH)D_3_ and 25(OH)D_2_ and consistently had 10–20% lower values than the specific LC‒MS reference methods used at other laboratories [22]. The total CV (intra- and inter-CV) of the method was 2.3% at 42.9 nmol/L and 1.97% at 145 nmol/L.

*S-Calcium*, albumin corrected (mmol/L), *S-alkaline phosphatase* (ALP) and *S-parathyroid hormone* (PTH) were measured. S-Calcium, S-ALP and S-PTH were measured with an Abbott Architect ci8200 (Abbott Laboratories, Illinois, USA) at the Department of Clinical Chemistry, Falun Hospital, Sweden.

*The diagnostic criteria for biochemical osteomalacia* were a S-25(OH)D concentration < 30 nmol/L, a PTH concentration > 6.9 pmol/L and an ALP concentration > 1.8 ukat/L [10]. The presence of clinical symptoms (grip weakness, leg weakness, a positive Trendelenburg test, fatigue and pain) was used to reaffirm the diagnosis [10].

### Study size

As one of the biomarkers for osteomalacia 25(OH)D was used. In a power calculation to find a difference of 25(OH)D of 20 nmol/L between Swedish and Somali women, with alpha error = 0.05 and beta value of 0.94 a minimum of 30 Swedish women and 30 Somali women were needed. Due to expected losses we aimed to include twice as many participants and to ensure power we aimed for a ratio of approximately 1 to 1.5 (Somali/Swedish).

### Statistical analysis

Summary statistics were calculated using standard methods. The analyses were performed using the SAS program package (version 9.4, SAS Institute, Cary, NC.). 

## Results

The characteristics of the women in both groups are presented in Table 1. Compared with Swedish women, Somali women wore more veiled clothing, had less exposure to sunlight and had given birth to more children. They also had a lower intake of milk, cheese, and fatty fish.

**Table 1:** Characteristics of Somali and Swedish women during pregnancy or lactation. Mean (standard deviation), median (interquartile range) and number (%) are presented

	Origin				
	Somalia		Sweden		
Variable	n^a^		n^a^		p=^b^
Veiled clothing^c^	51	51 (100)	69	0 (0)	<0.001
Outdoors^d^	51	30 (58.8)	69	66 (95.6)	<0.001
Sunbathing^e^	51	14 (27.4)	69	66 (95.6)	<0.001
Sun holiday in winter^f^	51	1 (2.0)	69	11 (15.9)	0.012
Time in Sweden (yr)	51	3.6 (2.6)	-	-	-
Time to inclusion^g^ (wks)	48	16.6 (17.3)	68	10.0 (6.8)	<0.001
Included in pregnancy	52	19 (36.5)	71	47 (66.2)	0.001
Weeks of gestation	19	11.3 (6.5)	45	11.4 (7.5)	0.948
Included in lactation	52	33 (63.5)	68	24 (35.3)	0.002
Time after delivery (wks)	29	25.0 (20.0)	26	7.0 (3.9)	<0.001
Nulliparous	52	6 (11.5)	71	17 (23.9)	0.08
Parity	52	3 (4)	71	1 (1)	<0.001
Age (yr)	52	28.3 (6.4)	71	30.6 (5.1)	0.024
Education (yr)	43	3.3 (3.4)	30	13.8 (2.5)	<0.001
Weight before pregnancy (kg)	21	61.4 (13.8)	64	68.4 (13.5)	0.045
Height (m)	49	1.61 (0.05)	69	1.67 (0.06)	<0.001
Physical activity (>twice/week)	51	25 (49)	71	33 (46)	0.140
Milk consumption (dL/day)	52	2 (2)	71	4 (3)	<0.001
Fat fish consumption (times/week)	52	0 (1.5)	71	1.5 (0)	0.004
Cheese consumption (times/week)	52	1.5 (5.25)	71	4 (2.5)	<0.001

^a^ Effective responses

^b^ The probability of no difference between the groups was tested by χ2-test, Mantel-Haenszel χ2-test or Wilcoxon’s non-parametric test

^c^ At least, long sleeved arms and legs and covered hair and neck, during summer in Sweden

^d^ Outdoor ≥30 minutes every day during summer in Sweden

^e^ Sunbathing face, neck and forearms during summer in Sweden

^f^ Sunbathing on lower latitudes

^g^ Weeks of gestation or weeks from delivery to date of study inclusion

During pregnancy, both Swedish and Somali women were included in approximately 11 weeks of gestation. During lactation, Somali women were included later in the postpartum period than Swedish women (25 weeks vs 7 weeks respectively). Swedish women were slightly older, had more years in education, were taller and weighed more prior to their pregnancies.

Somali women were found to have lower levels of 25 hydroxy vitamin D levels, averaging in the deficiency range, compared to Swedish women, with average vitamin D levels in the insufficiency range (16nmol/L vs. 49nmol/L respectively). Among Somali women 51/52 (98%) were insufficient and 47/52 (90%) were deficient and among Swedish women 47/71 (54%) were insufficient and 7/71 (10%) were deficient (data not shown). PTH levels are subsequently higher in Somali women compared to Swedish women (12.5pmol/L vs. 4.64pmol/L respectively) as are ALP levels (1.98ukat/L vs. 1.48ukat/L). Serum albumin corrected calcium levels were comparable. With respect to muscle strength assessment, Somali women had a weaker grip, greater difficulty in performing squats and more Somali women had a positive Trendelenburg test indicating proximal muscle weakness involving the hip abductors. Also, the Somali women reported almost double the level of pain intensity compared to Swedish women (Table 2).

**Table 2:** Biochemical findings, physical signs and symptoms among Somali and Swedish women during pregnancy or lactation. Mean (standard deviation), median (interquartile range) and number (%) are presented

Variable	n^a^		n^a^		p=^b^
25-hydroxy vitamin D (nmol/L)	52	16.0 (9.6)	71	49.4 (18.4)	<0.001
Parathyroid hormone (pmol/L)	52	12.5 (10.8)	70	4.64 (2.56)	<0.001
Alkaline phosphatase (μkat/L)	52	1.98 (1.38)	71	1.48 (0.69)	0.018
Calcium, albumin corrected (mmol/L)	52	2.28 (0.12)	71	2.30 (0.08)	0.112
Grip strength^c^ (N)	52	198.8 (58.1)	71	318.0 (66.6)	<0.001
Squat^d^	51	1 (1 to 2)	70	0 (0 to 0)	<0.001
Trendelenburg’s test^e^	52	1 (1 to 2)	71	0 (0 to 0)	0.001
Fatigue^f^ VAS (mm)	52	45.2 (39.8)	71	52.0 (22.8)	0.271
Pain^f^ VAS (mm)	51	29.4 (39.2)	71	15.3 (19.7)	0.021

^a^ Effective responses

^b^ The probability of no difference between the groups was tested by χ2-test, Mantel-Haenszel χ2-test or Wilcoxon’s non-parametric test

^c^ Peak grip strength measured with hand dynamometer (Grippit®)

^d^ Squat performed once, 0=without difficulty, 1=with difficulty (e.g. support by hands on knees), 2=with great difficulty (e.g. support by hands on furniture) or unable to arise from the squatted position

^e^ 0=maintained pelvis horizontal to the floor, 1=unable to maintain pelvis horizontally to the floor, 2=unable to stand on one leg because of muscle weakness

^f^ Fatigue = experienced tiredness during past month marked on a 100 mm long visual analogue scale with not tired at all = 0 mm and unbearably tired = 100 mm

^e^ Experienced pain now, regardless of location, marked on a 100 mm long visual analogue scale with no pain at all = 0 mm and unbearably pain = 100 mm

Of all Somali women, 34.6% met the biochemical diagnostic criteria for osteomalacia with 30.8% of whom also had signs or symptoms reaffirming the diagnosis. On the other hand, only 1.4% of Swedish woman met the criteria for biochemical osteomalacia (Table 3). Among women with affirmed biochemical osteomalacia diagnosis, none was explained by differences in hemoglobin, systolic blood pressure and blood glucose (data not shown).

**Table 3:** Osteomalacia diagnosis and proportions of its reaffirmation by signs or symptoms among Somali and Swedish women during pregnancy or lactation. Proportions in numbers and % are presented

	Origin				
		Somalia		Sweden	
Variable	n^a^		n^a^		p=^b^
Biochemical diagnosis^c^	52	18 (34.6)	71	1 (1.4)	<0.001
Reaffirmed biochemical diagnosis^d^	51	16 (30.8)	71	1 (1.4)	<0.001
Signs or symptoms reaffirming the diagnosis					
Grip weakness^e^	52	9 (17.3)	71	1 (1.4)	0.002
Leg weakness^f^	51	12 (23.5)	71	0	<0.001
Positive Trendelenburg’s test^g^	52	5 (9.6)	71	0	0.012
Fatigue^h^	52	6 (11.5)	71	0	0.005
Pain^i^	51	3 (5.9)	71	1 (1.4)	0.17

^a^ Effective responses

^b^ The probability of no difference between Swedish and Somali women was tested by χ2-test and Fischer’s exact test

^c^ S-25-hydroxy vitamin D <30.0 nmol/L and S-parathyroid hormone >6.90 pmol/L and S-alkaline phosphatase >1.80 μkat/L

^d^ Reaffirmed by either grip weakness, leg weakness, positive Trendelenburg’s test, fatigue or pain

^e^ Peak grip strength less than -2 standard deviations of peak grip strength measured with hand dynamometer (Grippit®)

^f^ Inability to perform one squat without difficulty, corresponding to the 75^th^ percentile

^g^ Positive Trendelenburg’s test (sign of weakness of hip abductors), corresponding to 90^th^ percentile

^h^ Fatigue = experienced tiredness during past month of >70 on a 100 mm visual analogue scale, corresponding to 75^th^ percentile

^i^ Experiencing pain intensity, regardless of location, >28mm on a 100 mm visual analogue scale, corresponding to 75^th^ percentile

The proportion of Somali women who were reaffirmed to have osteomalacia during pregnancy was 5/20 (25.0%), and that during lactation was 11/32 (34.4%), derived from study data, not shown in Table 3. The Swedish woman with reaffirmed osteomalacia diagnosis was in lactation.

## Discussion

### Key findings

One third of Somali women were diagnosed with biochemical osteomalacia, whereas one Swedish woman fulfilled the diagnostic criteria. The biochemical osteomalacia was reaffirmed by signs or symptoms in almost every woman. The large differences in sunlight exposure and differences in nutrition suggest that vitamin D plays an important role in the development of biochemical osteomalacia. In addition, almost all Somali women showed vitamin D deficiency and the characteristic biochemical pattern of bone degradation. Notable one tenth of Swedish women had vitamin D deficiency. 

### Interpretation

Because pregnancy and lactation typically suppress PTH through increased calcitriol and PTHrP levels, elevated PTH under these conditions likely reflects a pathological disturbance in mineral metabolism rather than normal physiology [13–17]. Mean PTH levels were twice the upper reference limit among Somali women and nearly three times higher than those observed among Swedish women, and mean ALP levels exceeded the upper reference limit. Notably, mean calcium levels were within the normal range in both groups. Therefore, in the presence of insufficient vitamin D levels, our results suggest that PTH and ALP should also be assessed.

The very low vitamin D levels observed among Somali women appear to be associated with extrinsic factors such as limited cutaneous UV exposure and low dietary intake of vitamin D and calcium. These factors render immigrants from lower to higher northern latitudes particularly vulnerable to vitamin D deficiency [8, 9]. Within this population, vitamin D and calcium supplementation is essential to prevent osteomalacia.

During pregnancy, the current gold standard for diagnosing osteomalacia, i.e. tetracycline-labeled bone biopsy and radiological assessment, is contraindicated. Therefore, a non-radiological, non-invasive diagnostic approach incorporating measurements of 25(OH)D, PTH, ALP, and functional assessments represents a clinically pragmatic alternative [10]. 

### Comparison with existing literature

Few contemporary studies have explored osteomalacia during pregnancy or lactation, largely due to the impracticality and ethical constraints of bone biopsy and radiological investigations in pregnancy. Overall, moving from lower to higher latitudes is associated with an increased risk of vitamin D deficiency due to reduced sunlight exposure [23, 24]. Our findings align with these studies as well as an earlier Scandinavian stud reporting severe vitamin D deficiency in Somali women [9]. To our knowledge, this is one of the first studies to estimate osteomalacia prevalence in a pregnant and postpartum immigrant and native population using a structured protocol [10]. 

### Clinical implications

Individuals with limited ultraviolet (UV) exposure, such as those who wear veiled clothing or have highly pigmented skin, as well as individuals with malabsorption, or those following vegetarian or vegan diets while avoiding milk products, should be considered for assessment of serum 25(OH)D concentrations. Diffuse unexplained pain, fatigue, or muscle weakness should also raise suspicion of possible vitamin D deficiency, particularly in individuals of southern origin or those who wear veiled clothing. When vitamin D deficiency is identified, evaluation of PTH, ALP, and serum calcium is recommended to characterize the metabolic impact.

In women with confirmed vitamin D deficiency, supplementation with vitamin D and calcium is essential to prevent the development of osteomalacia. Among pregnant or lactating women with established osteomalacia, adequate replacement of both vitamin D and calcium constitutes the cornerstone of therapy. 

### Strengths and limitations

Strengths of the study include standardized biochemical assays, validated functional tests, systematic assessment of dietary and lifestyle factors, and data collection confined to late spring, thereby minimizing seasonal variation in vitamin D status. Participants were enrolled through standard antenatal care, and review of clinical records allowed exclusion of untreated endocrine or neurological conditions that could influence muscle function.

The clinical interpretation of our results strengthens by the structured, non-radiological, non-invasive protocol for biochemical osteomalacia, i.e. the combination of biochemical markers and functional assessments.

Limitations include retrospective inclusion, reliance on self-reported dietary and symptom data. and a small number of Swedish women fulfilling criteria. These factors warrant cautious interpretation of subgroup differences but do not diminish the overall pattern observed.

We did not measure magnesium, zinc, copper, manganese, or vitamin B12 directly, which may theoretically influence ALP or PTH responses. However, hemoglobin measurements, serum phosphate levels dietary information, antenatal screening routines, and the Swedish social support system all reduce the likelihood of severe micronutrient deficiencies explaining the findings. Women meeting osteomalacia criteria exhibited the characteristic vitamin D deficiency pattern rather than hypophosphatemic profiles.

Differences in timing of inclusion between pregnant and lactating women introduced hormonal variability. However, pregnancy and lactation both typically suppress PTH, the elevated values of PTH observed are unlikely to be attributable solely to physiological stage. 

### Implications for practice and future research

Given the high global prevalence of vitamin D deficiency [25, 26] and the practical and ethical constraints of current diagnostic gold standard, there is a need for validated, non-invasive diagnostic criteria suitable for maternity and primary care globally. Our findings support the feasibility of such an approach and highlight the importance of identifying women with clinically significant vitamin D deficiency early in pregnancy and postpartum. Future studies should validate diagnostic thresholds for osteomalacia in diverse populations and investigate whether targeted supplementation or lifestyle modification can improve maternal musculoskeletal health and reduce adverse outcomes. 

## Conclusions

Women of Somali origin living at high altitudes are at high risk for vitamin D deficiency osteomalacia, but Swedish women may also suffer from this disease. Women at risk of osteomalacia with clinical signs and symptoms should be examined for the levels of vitamin D, parathyroid hormone, alkaline phosphate and not only calcium. Establishing internationally accepted criteria for a noninvasive clinical diagnostic procedure for osteomalacia is imperative.

## Supplementary Information

Supplementary Material 1.

Supplementary Material 2.

## Data Availability

The data used for this study are available in the supplementary file ‘Data osteomalacia’. The more sensitive questionnaire data are available from the corresponding author upon reasonable request.

## Abbreviations

ALP: Alkaline phosphatase
CI: Confidence interval
DAG: Directed Acyclic Graph
PTH: Parathyroid hormone
25(OH)D: 25-hydroxy vitamin D
