# Supplementary material for: Biochemical osteomalacia during pregnancy or lactation: an observational study in Sweden
Source: BMC Pregnancy Childbirth. 2026 Feb 24;26:220. doi: 10.1186/s12884-026-08848-1 (PMC12955220; doi:10.1186/s12884-026-08848-1)
Supplement: Supplementary file 1 — Supplementary Material 1. [file 12884_2026_8848_MOESM1_ESM.pdf]

Code:…………………………………………………Date……………………………………….................

Questionnaire VITAMIN D

1. When were you born? Year….

2a. Are you pregnant?
☐ no
☐ yes. If yes, when are you going to give birth? ………………

b. Are you breastfeeding?
☐ no.
☐ yes. If yes, how long have you been breastfeeding? ………… year …………. months.

3a. In what country are you born? ..……………………………………………………….

b. In what country is your mother born? ……………………………………………………..

c. In what country is your father born? ………………………………………..…………..

4a. How long have you been living in this country? …………. years.

b. Did you live in another country before you came here?

☐ no
☐ yes. If yes, what country? ………………………………… For how long? .... years.

5a If you have been living in another country. Did you have any medical illness or disease?

Country?... Disease? ……………………………………

....

b. Do you have any symptoms since then? Which ones?

…………………………………………………………………………………………….

---

6. Do you have any disease(-s) now? Which one(-s)?

…………………………………………………………………………………………….

…………………………………………………………………………………………….

7a What medicine do you take every day (if any)? Which and for what reason?

1. .... 2. ……………………………………………

3.……………………………………….. 4. ……………………………………………

5. ……………………………………… 6. …………………………………………….

b. What medication to you take occasionally?

1. …………………………… 2. …………………………… 3. ……………………….

4. ……………………………

---

8. Do you take supplements, vitamins, or naturopathic drugs?
☐ no
☐ yes
If yes, what kind and for what reason? 1……………………………………………………..
2 ………………………………….. 3…………………………………………………….
4 ………………………………….. 5……………………………………………………..

---

9. Do you take calcium supplements with or without vitamin D?
☐ no
☐ yes
If yes, what kind and how many per day? ……………………………………………………

---

10. Have you had any fractures?
☐ no
☐ yes

Where?
☐ hip Year.......
☐ vertebrae Year…….
☐ wrist Year.…….
☐ other location……………………………………………………………………………..

11a. How is your health right now? Mark with a cross on the line.

Worst thinkable ◆——————————————◆ Best imaginable
Really bad Really good

11b. Do you have pain in your body right now? If yes, mark the location on the figure.

Pain drawing

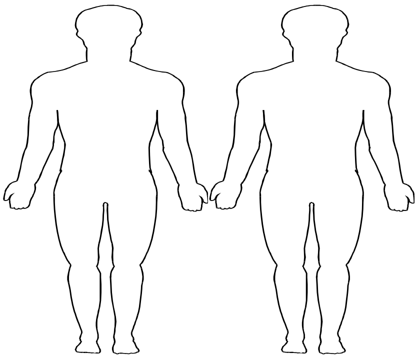

The pain position: Superficial ……… Deep……….
Pain character: Burning… Biting… Cutting… Stabbing… Pounding… Painful… Pressing…

Are you in pain right now? If yes, how painful is it? Mark with a cross on the line.

No pain ◆————————————————◆ Intolerable pain

---

12. Life habits
Do you smoke?
☐ yes
☐ no, never smoked
☐ smoked earlier. Stopped year ……….

---

13. Approximately how many deciliters or glasses of milk do you drink every day?
.... deciliter or …………glasses.

---

14. How often do you eat cheese?
☐ never
☐ maximum 1 – 2 times/week
☐ 3 – 5 times/week
☐ 6 – 7 times/week

---

15. How many times per week do you eat salmon or other fatty fish?
☐ never
☐ 1 – 2 times/week
☐ 3 or more times/week

---

16. Do you drink alcohol (vine, sprits, strong beer) minimum once per week?
☐ no
☐ yes

17. Do you exercise (minimum half an hour of promenade or gymnastics)?
☐ seldom or never
☐ 1 – 2 times/week
☐ minimum 3 times/week

---

18. Are you outdoors in daytime a minimum of 30 minutes every day in the summer?
☐ no
☐ yes

19a. Do you sunbathe your face, neck and forearms during the summer?
☐ no
☐ yes

b. Do you sunbathe at least one week in a sunny country during the winter?
☐ no
☐ yes

---

20. How many children have you given birth to? ……………. children.

---

21. Did you breastfeed your children?
☐ no
☐ yes
If yes, how long? .... months.

---

22. How do you dress during the summer?
☐ short-sleeved blouses/jumpers/T-shirts and short skirts/dresses
☐ long-sleeved blouses/jumpers ang long pants/dresses without protection for face and hands
☐ I wear veiling clothing or burka

Functional tests

1. Have you felt tired this last month? Mark with a cross on the line.

Not tired at all ◆————————————◆ Intolerable tiredness Can´t do anything

2. Are you dizzy or have bad balance? Mark with a cross on the line.

No dizziness at all ◆————————————◆ Extremely dizzy Can´t stand up

3. Do you feel irritated? Mark with a cross on the line.

Not at all ◆————————————◆ Angry all the time

4. Did you feel sad the last four weeks? Mark with a cross on the line.

Not sad at all ◆————————————◆ Very sad. Crying all time

5. Do you feel week in your arms and legs? Mark with a cross on the line.

Not at all ◆————————————◆ Very weak. Can´t do anything.

6.Squatting

a. Can you squat and rise?
b. Can you squat and rise from a chair without holding on to anything?
c. Can you stand on one leg? Please, show how you do it.

(Grading
A: Without problem B: With difficulty, needs to hold on to something C: Needs to hold on to someone)

7 Trendelenburgs test
Interpretation of Trendelenburgs test (see methods)

8 Grip strength
Measurements (see methods)

Were you on sick leave from job or school during your pregnancy?

☐ no
☐ yes. Why………………………………………………. how long………. months

Antropometric measurements:

Height: …………. cm.

Weight (without outer clothing and shoes): …………… kg

Waistline: …………… cm.

BMI: …………….

Blood pressure: ……………. mm/Hg
